# Changing Staphylococcus aureus Resistance in Atopic Dermatitis: Implications for Topical Antimicrobial Use From a Regional Study in the United Kingdom

**DOI:** 10.7759/cureus.97141

**Published:** 2025-11-18

**Authors:** Bilal Amer Mian, Aiden J Plant

**Affiliations:** 1 Department of Microbiology, Walsall Healthcare NHS Trust, Walsall, GBR; 2 Department of Microbiology, Black Country Pathology Services, Walsall, GBR

**Keywords:** antimicrobial resistance, atopic dermatitis (ad), dermatology trends, methicillin resistant staphylococcus aureus, staphylococcal aureus

## Abstract

Background: *Staphylococcus aureus* plays a central role in the pathogenesis and exacerbation of atopic dermatitis, with superinfection contributing to disease severity and treatment challenges. As antimicrobial resistance evolves, understanding local susceptibility patterns is essential for dermatological practice and antibiotic stewardship.

Methods: A retrospective analysis was conducted of *Staphylococcus aureus (S. aureus)* isolates from skin and soft tissue infections in the Black Country region of the United Kingdom between 2019-2024, with community isolates obtained from general practice and hospital isolates from both inpatient and outpatient settings. Antimicrobial susceptibility was compared between adults and children (0-17 years), with methicillin-resistant *S. aureus* (MRSA) defined by flucloxacillin/oxacillin resistance. Statistical comparisons were made using Chi-squared testing.

Results: Among 30,160 community isolates, MRSA prevalence was 7%. Hospital-based *S. aureus* isolates (n=2,733) demonstrated 11% MRSA prevalence. Paediatric isolates (n=272) showed 10% MRSA prevalence, which were not significantly different from adults (p=0.47). Fusidic acid resistance, a key concern in dermatology due to its frequent topical use for atopic dermatitis, was significantly higher in children (21% vs 14%, p=0.002). Adults demonstrated greater resistance to erythromycin, clindamycin, and tetracycline.

Conclusion: This study highlights changing staphylococcal epidemiology with concerningly high MRSA and fusidic acid resistance in dermatological isolates. Given the frequent use of topical fusidic acid in atopic dermatitis, its role in management should be reconsidered. The findings underscore the need for dermatology-led antimicrobial stewardship to mitigate escalating community resistance in* S. aureus* infections.

## Introduction

Atopic dermatitis is a common inflammatory skin disease predominantly affecting children, with up to one in five children afflicted [1]. The underlying pathology is driven by skin barrier dysfunction, and treatment is centred on emollients, improving skin barrier function, with topical corticosteroids utilised to reduce inflammation [2,3]. 

Complications include *Staphylococcus aureus* (*S. aureus)* colonisation; subsequent super-infection of atopic dermatitis is a leading cause of morbidity in this cohort, whereby treatment of mild-moderate infection is treated topically, whilst severe infection requires systemic antimicrobial therapy [4]. 

Topical fusidic acid is licensed for the treatment of staphylococcal skin and soft tissue infections in the United Kingdom, and has been considered the mainstay of treatment for mild-moderate infective exacerbations for decades [5]. Given that antimicrobial resistance is an increasing global concern, it is reassuring that a recent meta-analysis found no significant change in the global antimicrobial susceptibility of *S. aureus* causing infections in patients with atopic dermatitis between 1985 and 2023 [4,6]. Whilst global epidemiology is reassuring, local rates of antimicrobial resistance must be consistently considered to ensure regional prescribing guidance that is applied to the population it serves. 

Based on this we conducted a study to review local susceptibility patterns of *S. aureus* within the Black Country region of the United Kingdom, where our district general hospital is based. Our aim was to assess the prevalence of *S. aureus* resistance, including MRSA, in children compared with the general population, given that children bear the highest burden of atopic dermatitis and are therefore at increased risk of skin dysbiosis and disproportionate pathology secondary to *S. aureus*.[1,7].

## Materials and methods

To estimate the prevalence of MRSA in the general population, we conducted a retrospective observational study analysing laboratory surveillance data from 2019 to 2024. The study assessed MRSA prevalence among both community and hospital isolates. Every swab yielding *S. aureus* from across the Black Country region was obtained, and methicillin resistance was determined based on their resistance to ​​​​​​flucloxacillin/oxacillin (fluclox/oxacillin), according to EUCAST (European Committee on Antimicrobial Susceptibility Testing) breakpoints using automated susceptibility testing (bioMérieux VITEK2).

Swabs yielding *S. aureus* from general practices across the Black Country were utilised as a means of modelling community MRSA prevalence, with swabs from all secondary care settings excluded. 

Data were obtained from the laboratory information management system for adults (≥18 years) and children (0-17 years), all swab results from all skin and soft tissue infections yielding S. aureus in both the inpatient and outpatient settings between 2019 to 2024 from our local district general hospital were included. From amongst the paediatric samples, those taken from the post-natal and midwifery wards were excluded to reduce the risk of bias introduced from maternal flora. The percentage sensitivity of isolates to different antibiotics was calculated, determined according to EUCAST breakpoints and VITEK2 automated susceptibility testing; MRSA prevalence was based on resistance to fluclox/oxacillin for both the paediatric and adult populations. Statistical analysis was carried out using 2x2 Chi-square analysis; statistical significance was determined between the general population and paediatrics, with a p<0.05 indicating significance.

## Results

Swabs culturing *S. aureus* from community-based samples totalled 30,160 isolates; of these 93% (27,942) were fluclox/oxacillin-sensitive, with 7% (2,218) deemed fluclox/oxacillin-resistant, accounting for an MRSA prevalence within the general population of 7%. Cumulative prevalence of *S. aureus *from hospital-based samples totalled 2,733 isolates.

Of these 89% (2,422) were fluclox/oxacillin-sensitive, with 11% (299) deemed fluclox/oxacillin-resistant, accounting for an MRSA prevalence within the hospital population of 11%. A total of 272 swabs from children cultured *S. aureus*; 90% (246) isolates were found to be fluclox/oxacillin-sensitive and 10% (26) were found to be fluclox/oxacillin-resistant. There was no statistically significant difference in prevalence of fluclox/oxacillin-resistant *S. aureus* between adults and children (p=0.47, 95% CI 0.58-1.25).

*S. aureus* resistance to other antibiotics is shown in Table 1. Children were more likely to harbour fusidic acid resistance (21%, n=56/272) compared to the general population (14%, n=377/2727; p=0.002, 95% CI 1.14-1.94). When comparing fluclox/oxacillin-sensitive and fluclox/oxacillin-resistant isolates and fusidic acid susceptibility, there was no significant difference, with fusidic acid resistance in 8% (n=17) MRSA isolates and 16% (n=9) MSSA isolates (p=0.63, 95% CI 0.46-1.23) respectively. There was no difference in resistance to trimethoprim between the general population and children, with 18% resistance in both groups (p=0.976, 95% CI 0.71-1.41). Resistance to erythromycin, clindamycin, and tetracycline was significantly higher in the general population than in children, with rates of 27% (734/2725) vs 21% (56/272, p=0.02; 95% CI 0.53-0.93), 25% (690/2733) vs 15% (41/272, p<0.001; 95% CI 0.38-0.72), and 13% (358/2727) vs 6% (16/272, p=0.001; 95% CI 0.25-0.68), respectively.

**Table 1:** Susceptibility data of S. aureus isolates in the general population vs children. * Global antibiotic sensitivity data for *S. aureus* isolates in patients with atopic dermatitis.

	General population				Children						
Antibiotic	Total tested (n)	Sensitive (n, %)	Resistant (n, %)	Percentage sensitivity (%)	Total tested (n)	Sensitive (n, %)	Resistant (n, %)	Percentage sensitivity (%)	Chi Square	Difference in sensitivity (χ², p)	Global (pooled) sensitivity from meta-analysis (*)
Clindamycin	2733	2043 (75%)	690 (25%)	75	272	231 (85%)	41 (15%)	85	13.9	<0.001	0.79
Erythromycin	2725	1990 (73%)	734 (27%)	73	272	216 (79%)	56 (21%)	79	5.1	0.02	0.73
Fluclox/oxacillin	2721	2422 (89%)	299 (11%)	89	272	246 (90%)	26 (10%)	90	0.5	0.47	0.85
Fusidic acid	2727	2350 (86%)	377 (14%)	86	272	216 (79%)	56 (11%)	79	9.2	0.002	0.80
Tetracycline	2727	2369 (87%)	358 (13%)	87	272	256 (94%)	16 (6%)	94	11.9	0.001	0.94
Trimethoprim	1744	1427 (82%)	317 (18%)	82	199	163 (82%)	36 (18%)	82	0.001	0.976	0.97

## Discussion

The prevalence of MRSA colonisation within the general population of the Black Country is 7%, compared to the commonly cited UK average of 1-3% [8]. The Black Country has high levels of socioeconomic deprivation, as well as diverse multiculturalism, with the potential to continually propagate community MRSA transmission to exceed the national baseline [9,10]. Whilst we note the prevalence of MRSA colonisation between the general population and children is not significantly different, the general population is more likely than children to harbour resistance to macrolides, lincosamides, and tetracyclines. This supports the understanding that cumulative exposure to antimicrobials increases the risk of developing resistance, making older age a significant risk factor for infections caused by antimicrobial-resistant organisms [11]. 

Atopic dermatitis is associated with *S. aureus* superinfection [4], and in the UK treatment is typically topical, with fusidic acid recommended as second-line following topical hydrogen peroxide [12]. Importantly, overuse of antibiotics is associated with resistance. We have demonstrated that frequent topical fusidic acid use in childhood atopic dermatitis should be considered a risk factor for fusidic acid resistance compared with the general population. This mirrors the findings of meta-analysis, which described a pooled fusidic acid sensitivity of 80% in those with atopic dermatitis (compared to 79% in our cohort) [1]. It has been identified since 1962 that low-dose and monotherapy exposure of staphylococci to fusidic acid leads to the rapid emergence of resistance, and despite the advice of using topical fusidic acid with caution, this has largely been ignored [13].

For over 20 years, concerns surrounding fusidic acid resistance within patients with dermatological conditions in the UK have been present, yet despite this, fusidic acid usage rates remain high [14]. Concerningly, in the UK, topical fusidic acid is accessible without an individual patient prescription via a patient group directive (PGD), including Pharmacy First services [15]. 

Given fusidic acid resistance now exceeds 20% in this population, prescribers should be much more cautious with its ongoing use, and policymakers should revisit its location in empiric formularies and availability via PGDs. Notwithstanding, high rates of resistance also preclude the use of this important antimicrobial in the systemic treatment of deep-seated staphylococcal infections. It is essential that future development of agents to treat infective exacerbations of atopic dermatitis, avoid the use of antimicrobial agents, which can propagate resistance, in preference for topical disinfectants. 

Equally concerning is resistance to commonly used antimicrobials, with erythromycin, clindamycin, and trimethoprim exceeding 10% resistance, with local resistance exceeding that of the global meta-analysis for trimethoprim in particular. It is important that guideline developers consider their local epidemiology when developing guidance. Troublingly, within the Black Country, children with atopic dermatitis and *S. aureus* super-infection alongside a penicillin allergy have only tetracycline-based agents as a reliable, commonplace, and systemic treatment option. However, this agent is limited by its relative contraindication for younger children due to concerns for dental discolouration and impaired bone growth, leaving few alternative oral options [16]. Prevention of staphylococcal super-infection in those with atopic dermatitis should be considered as a priority, avoiding the need for both topical and systemic antimicrobial therapy, yet current evidence for a robust *S. aureus *decolonisation regimen is lacking, often failing to eradicate long-term carriage [8]. 

Limitations

Whilst this study reflects one of the largest regional datasets of *S. aureus *resistance patterns relative to dermatological practice in the UK, it also has notable limitations; it is observational in nature, suggesting correlation but not causation. Children were compared against the general population, rather than adults specifically, meaning some resistance data will overlap. Data from children was used to infer the susceptibility profile of *S. aureus* from those with atopic dermatitis based on the probability of the diagnosis in this cohort, rather than specifically from children with atopic dermatitis, which may reflect sampling bias. The data has not been controlled for potential factors that may impact resistance, such as previous antibiotic use, co-morbidities, or socioeconomic status. Finally, the data is highly specific to the local population of the Black Country and should not be considered generalisable. 

## Conclusions

To conclude, whilst MRSA colonisation is no more common in children with atopic dermatitis than in the general population, children harbour high rates of resistance to fusidic acid and, therefore, the role of this topical antimicrobial needs to be reconsidered. MRSA prevalence in both adults and children is locally higher than the national average, and compared to global resistance rates, local resistance to clindamycin, trimethoprim, and tetracycline is concerningly high. 

These findings highlight the importance of dermatology-led antimicrobial stewardship to address the rising community resistance seen in *S. aureus* infections. Preventing *S. aureus* superinfection in atopic dermatitis should be prioritised, with further research efforts focused on identifying an effective *S. aureus *decolonisation regimen of the highest importance.
